# Use of routine healthcare data in randomised implementation trials: a methodological mixed-methods systematic review

**DOI:** 10.1186/s13012-023-01300-4

**Published:** 2023-10-02

**Authors:** Charis Xuan Xie, Lixin Sun, Elizabeth Ingram, Anna De Simoni, Sandra Eldridge, Hilary Pinnock, Clare Relton

**Affiliations:** 1 Wolfson Institute of Population Health, Queen Mary University of London, London, England UK; 2 School of Health and Related Research, University of Sheffield, Sheffield, England UK; 3 Department of Applied Health Research, University College London, London, England UK; 4 Asthma UK Centre for Applied Research, Usher Institute, The University of Edinburgh, Edinburgh, Scotland UK

**Keywords:** Routine healthcare data, Implementation science, Trials, Review

## Abstract

**Background:**

Routine data are increasingly used in randomised controlled trials evaluating healthcare interventions. They can aid participant identification, outcome assessment, and intervention delivery. Randomised implementation trials evaluate the effect of implementation strategies on implementation outcomes. Implementation strategies, such as reminders, are used to increase the uptake of evidence-based interventions into practice, while implementation outcomes, such as adoption, are key measures of the implementation process. The use of routine data in effectiveness trials has been explored; however, there are no reviews on implementation trials. We therefore aimed to describe how routine data have been used in randomised implementation trials and the design characteristics of these trials.

**Methods:**

We searched MEDLINE (Ovid) and Cochrane Central Register of Controlled Trials from Jan 2000 to Dec 2021 and manually searched protocols from trial registers. We included implementation trials and type II and type III hybrid effectiveness-implementation trials conducted using routine data. We extracted quantitative and qualitative data and narratively synthesised findings.

**Results:**

From 4206 titles, we included 80 trials, of which 22.5% targeted implementation of evidence-based clinical guidelines. Multicomponent implementation strategies were more commonly evaluated (70.0%) than single strategies. Most trials assessed adoption as the primary outcome (65.0%). The majority of trials extracted data from electronic health records (EHRs) (62.5%), and 91.3% used routine data for outcome ascertainment. Reported reasons for using routine data were increasing efficiency, assessing outcomes, reducing research burden, improving quality of care, identifying study samples, confirming findings, and assessing representativeness. Data quality, the EHR system, research governance, and external factors such as government policy could act either as facilitators or barriers.

**Conclusions:**

Adherence to guidance on designing and reporting implementation studies, and specifically to harmonise the language used in describing implementation strategies and implementation outcomes, would aid identification of studies and data extraction. Routine healthcare data are widely used for participant identification, outcome assessment and intervention delivery. Researchers should familiarise themselves with the barriers and facilitators to using routine data, and efforts could be made to improve data quality to overcome some of the barriers.

**Registration:**

PROSPERO CRD42022292321.

**Supplementary Information:**

The online version contains supplementary material available at 10.1186/s13012-023-01300-4.

Contributions to the literature

- This systematic review summarises key methodological characteristics of randomised implementation trials conducted using routine healthcare data and synthesises the reported rationales, facilitators, and barriers.
- We identified key gaps in the reporting of implementation trials and highlight the importance of adopting and adhering to existing guidelines on designing and reporting implementation studies, especially the need to harmonise the language in describing the implementation strategies and outcomes.
- We found rationales for using routine data were increasing efficiency, assessing outcomes, reducing research burden, improving quality of care, identifying study samples, confirming findings, and assessing representativeness. Data quality, the EHR system, research governance, and external factors such as government policy could act either as facilitators or barriers.

## Introduction

The randomised implementation trial (hereafter implementation trial) evaluates the effect of implementation strategies on implementation outcomes [1], in distinction to the effectiveness randomised controlled trial (RCT) which evaluates the impact of healthcare interventions on health-related outcomes. Implementation trials aim to promote the uptake of evidence-based interventions into practice by employing various strategies. For example, text messaging increases the adoption of influenza vaccination [2]. The Expert Recommendations for Implementing Change (ERIC) study [3] identified 73 commonly used implementation strategies, such as audit and feedback, financial incentives, and educational and training sessions. These were then mapped into categories relating to infrastructure change, implementation context, and stakeholders’ support and management [4]. Implementation outcomes are key indicators of the implementation process; they are also intermediate outcomes in relation to health outcomes (e.g. biomarkers, quality of life) [5]. Common implementation outcomes include the adoption of evidence-based interventions [2], the quality of programme delivery (fidelity) [6], the feasibility and acceptability of implementing interventions in a given context [7]. Proctor and colleagues conceptualised implementation outcomes into eight categories, including acceptability, adoption, feasibility, fidelity, appropriateness, penetration, sustainability, and costs [5]. Implementation trials may also report the effect on health-related outcomes. These types of trials have been described as effectiveness-implementation hybrid trial designs [8].

Routine healthcare data (hereafter routine data), obtained from electronic health records (EHR), administrative databases, and registries [9], are not collected for research purposes but are increasingly used for research, for example in the execution of RCTs [10]. The use of routine data in RCTs is deemed a novel trial design to improve the efficiency and effectiveness of RCT delivery, and the value of routine data potentially manifests in various ways [11, 12]. For example, Reeves and colleagues evaluated a weight loss intervention for women following treatment for breast cancer, for which 90 obese participants were recruited from a state-based cancer registry [13]; Sandner and colleagues assessed the effects of a targeted home visiting programme, in which they used administrative data from the German public health insurance system to measure the maternal mental health outcomes [14]; and Downing and colleagues evaluated an EHR-based clinical decision support alert for improving severe sepsis treatments [15].

To address the growing interest in employing routine data in RCTs and to improve the reporting quality, the Consolidated Standards of Reporting Trials extension for randomised controlled trials conducted using cohorts and routinely collected data (CONSORT-ROUTINE) has been developed [10]. The CONSORT-ROUTINE team reviewed published effectiveness RCTs using data from EHRs, registries, and administrative datasets and assessed those trials’ reporting transparency and completeness against the guideline [16–19]. There is, however, no review focused on how routine data are used in implementation trials. Therefore, the objectives of this review are to (1) describe the characteristics of implementation trial designs conducted using routine data, (2) investigate how routine data were used and reported in these implementation trials, and (3) explore the reported rationales, facilitators, and barriers of using routine data in implementation trials.

## Method

We conducted a methodology systematic review [20] to investigate the characteristics of randomised implementation trials using routine healthcare data. The review protocol was previously registered on PROSPERO CRD42022292321, and we report the results according to the PRISMA 2020 checklist [21] (see Additional file 1).

### Systematic searches

The literature search was conducted initially in two main databases: MEDLINE via Ovid and Cochrane Library. We originally planned to search Cochrane Methodology Registry, but it has not been updated since 2012 and does not support an advanced search function. We therefore searched Cochrane Central Register of Controlled Trials (CENTRAL) instead to capture all relevant implementation trials. The databases were searched from Jan 2000, due to the growing recognition of electronic health records and implementation science in the last two decades [22], to Dec 2021 (see Additional file 2 for search strategy). A manual search of citations was performed for unpublished and in-progress studies, and trial protocols were tracked in ClinicalTrials.gov, BMC ISRCTN registry, WHO International Clinical Trials Registry Platform, and Australian New Zealand Clinical Trials Registry.

### Definitions, inclusion, and exclusion criteria

Eligible RCTs had to be randomised implementation trials evaluating the effectiveness of implementation strategies to promote the uptake of evidence-based interventions/practices/programmes/treatments/services. Three types of implementation trials were considered: (1) implementation trials, where the goal was to assess the impact of implementation strategies only on implementation outcomes; (2) type II hybrid effectiveness-implementation trials, where the co-primary aims were to determine the impact of the implementation strategies on implementation outcomes as well as the effectiveness of the intervention on health outcomes [8]; and (3) type III effectiveness-implementation hybrid trials where the primary focus was on implementation outcomes with the secondary focus on the intervention outcomes [8].

The trials had to use any type of routine data (EHR, administrative dataset, registry) in either (1) trial participants identification and recruitment, (2) outcome ascertainment, (3) intervention delivery, or (4) any combination of these uses. The included trials had to be peer-reviewed articles written in English. Nested economic and process evaluations were excluded. Studies that exclusively focused on health economic outcomes or only reported long-term follow-up outcomes were also excluded. Conference abstracts and study protocols were not included, but we performed a citation search for full publications reporting trial outcomes. A detailed description of inclusion criteria is outlined in Table 1.

**Table 1:** Inclusion criteria and specifications

	Inclusion criteria	Definitions, specifications, and examples
Population	Any	Any population who received or were targeted by a healthcare or health promotion intervention
Implementation strategy^a^	• Single implementation strategy: involving one action or process • Multicomponent implementation strategy: comprising two or more single strategies	• Examples of commonly used single implementation strategies: mailed outreach, reminders, education and training sessions, etc • Examples of multicomponent strategies: electronic health record-based patient identification plus individualised mailed outreach, quality improvement programme [23], model of care [24], implementation theory, and frameworks [25]
Comparator	• Usual care • Active comparator	E.g. no implementation strategy or active implementation components
Outcomes	• An implementation outcome had to be a primary focus of the eligible trial • Secondary outcomes may include health-related outcomes (including biomarkers of diseases), process outcomes (e.g. the amount of time used in prescription), changes in health behaviour, and quality of life	Eight common implementation outcomes [5]: Acceptability Adoption (e.g. uptake of the cancer screening programmes) Appropriateness (e.g. appropriateness of implementing a weight management programme in low-income countries) Feasibility Fidelity (e.g. quality of programme delivery) Implementation cost Penetration (e.g. the spread of quality improvement programme) Sustainability (e.g. the maintenance of a health service in the long term) A full description of the eight implementation outcomes is provided in Additional file 3
Study designs	Any RCTs	Including parallel RCTs, cluster RCTs, stepped-wedge RCTs, pragmatic RCTs, adaptive design, hybrid effectiveness-implementation design
Healthcare settings	• Primary care • Inpatient • Secondary care • Other	Primary care service includes general and family practice, community care, pharmacy, dental and ophthalmic services Inpatient care provided in the hospital settings Secondary care service includes general internal medicine, general paediatrics, obstetrics and gynaecology, outpatient Other may include multiple settings
Evidence-based interventions^a^	The evidence is preferably supported by systematic reviews and meta-analyses or a number of effectiveness RCTs The evidence is expected to be cited in the trial background (e.g. introduction or method sections)	E.g. evidence-based treatment/medications/services, clinical guidelines, cancer screening programmes, health policy
Type of routine data	Electronic health record/electronic medical record/personal health record	E.g. primary care databases
	Administrative datasets	E.g. healthcare claims databases, private insurance databases
	Registries	E.g. cancer registry, birth/death registry, HIV registry
Use of routine data	Trial participants identification	E.g. EHR data were used to identify eligible patients over 65 years old with blood pressure > 140/90 mmHg
	Outcome ascertainment	E.g. data from the HIV registry were analysed to assess the proportion of patients screened for HIV
	Intervention delivery	E.g. reminders (as an implementation strategy) integrated into the electronic health record
Type of trials considered in this review	Implementation trial: the aim was to assess the implementation strategies solely on implementation outcomes, as opposed to hybrid designs	E.g. a trial evaluated the effectiveness of a telephone reminder to increase the uptake of mammography screening [26]
	Hybrid type II design: the co-primary aims were to determine the impact of the implementation strategies on implementation outcomes and the effectiveness of the health intervention on health outcomes	E.g. a trial examined the effectiveness of the intervention on diabetes and depression and concurrently tested an implementation strategy to increase the use and fidelity of the intervention [27]
	Hybrid type III design: the primary focus was on implementation outcomes, with the secondary focus on the intervention outcomes	E.g. a trial evaluated an implementation strategy bundle, with the primary outcomes on the adoption and sustainability of the evidence-based intervention and secondary outcomes on activities of daily living, pain, depression, falls, emergency department visits, and hospitalisations [28]

^a^Aligned with the StaRI reporting standards [29], we use the term “implementation strategy” throughout this review to describe the “intervention” in the included trials (noting that the majority of the implementation trials did not adhere to this terminology), and we use “health intervention” or “evidence-based intervention” to describe the interventions that the implementation trials intended to implement/deliver in the real world

### Data screening

CX screened titles and abstracts of all searched records; LS independently screened a random sample of 10% of all titles and abstract. Due to poor reporting of implementation trials [16–18, 30], we included all publications with potential eligibility at this stage, even if the trials did not specify they were implementation trials or if the trials did not explicitly describe the use of routine data in the abstract. Any disagreements were resolved by discussion and consensus.

Full texts screening was undertaken by two reviewers (C. X. and L. S.). Again, C. X. assessed all full texts, with a random sample of 10% independently screened by L. S. Discrepancies between two reviewers were resolved through consultation with a third reviewer (E. I.). Further disagreements were resolved in consensus meetings with four senior researchers in the review team (C. R., S. E., H. P., A. S.).

### Data extraction and analysis

Data extracted from all relevant papers addressed the following: (1) general study characteristics: authors, year of publication, country, setting, health condition, and type of randomisation (individual, cluster); (2) characteristics of trials: type of trials (implementation, hybrid type II, hybrid type III), implementation strategies, comparators, implementation outcomes, and evidence-based interventions; (3) characteristics of routine data: types (EHR, administrative datasets, registries), usage (participant identification, outcome assessment, intervention delivery or combinations), and data linkage; and (4) reported rationales, barriers, and facilitators of using routine data in those trials. Two reviewers (C. X. and E. I.) independently extracted data from four trials (5%, 4/80), compared findings, and agreed on the initial data extraction. C. X. then completed the remaining data extraction. Any uncertainties were discussed with other authors.

Data were extracted into an Excel spreadsheet for analysis by CX. Descriptive statistics were performed using Stata SE v17 to summarise the study characteristics, implementation strategies and outcomes, and the type and usage of routine data. Thematic analysis was conducted to synthesise the reported rationale, barriers, and facilitators.

## Results

**Fig. 1:**
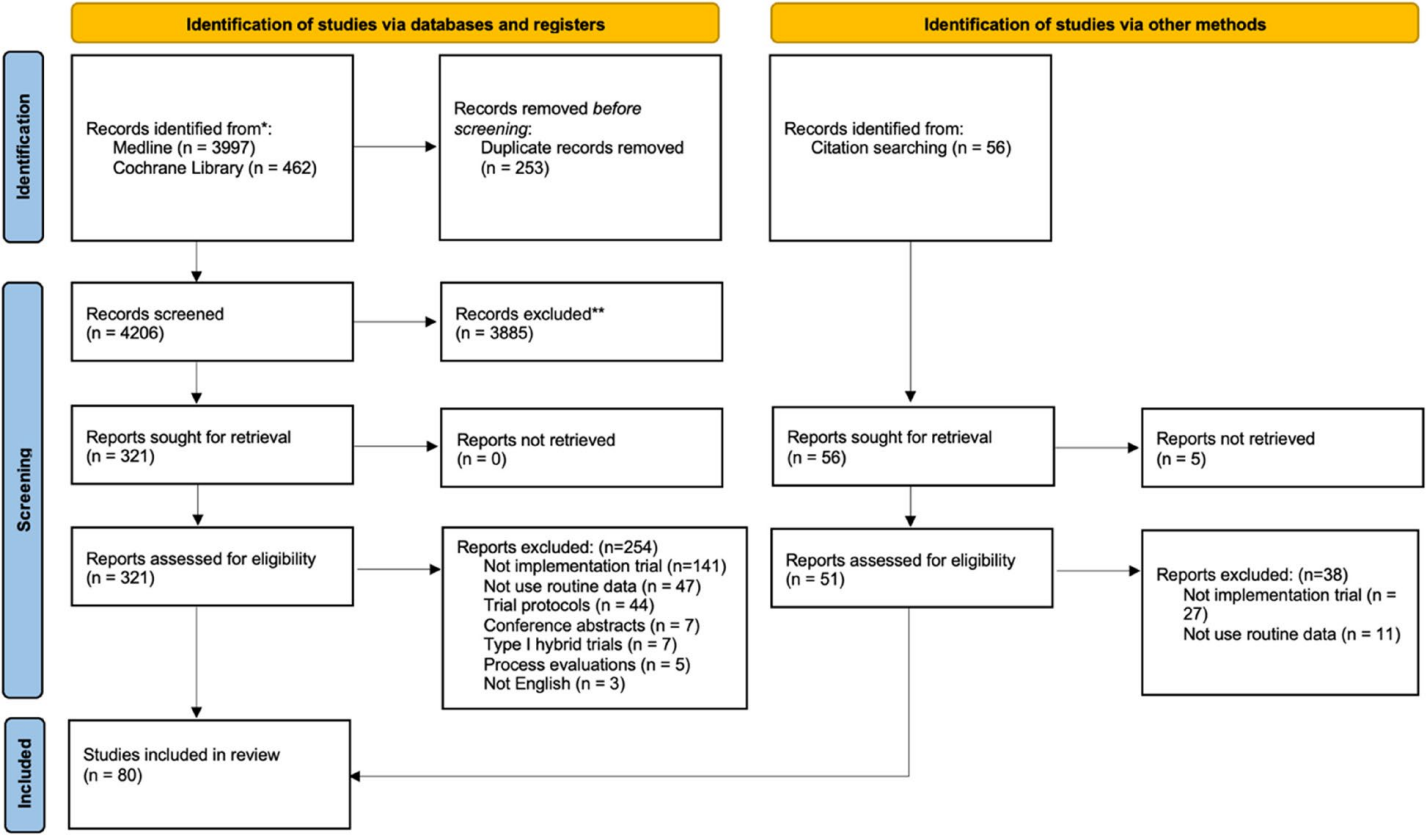
PRISMA 2020 flow diagram for new systematic reviews which included searches of databases, registers, and other sources

Overall, 4459 citations were retrieved from database searches. After the removal of duplicates, 4206 titles and abstracts were reviewed. Of those unique records, 3885 were excluded after the title and abstract review, and 254 were excluded after the full-text review. Sixty-seven eligible studies met inclusion criteria from database searches. We additionally found 13 trials via study protocols and citation tracking. Eighty studies were therefore included for data extraction. Figure 1 is the PRISMA flow diagram, and references for all eligible studies are provided in Additional file 4.

### Study characteristics

Table 2 presents the design and context of the 80 included implementation trials using routine data. Forty-three (53.8%) were cluster RCT designs, and 37 (46.3%) were individual designs. The implementation trials were predominantly conducted in North America (63.8%, *n* = 51) and in primary care settings (90.0%, *n* = 72). The most frequently researched medical areas were cardiovascular disease (15.0%, *n* = 12), general health (13.8%, *n* = 11), and cancer (13.8%, *n* = 11).

**Table 2:** Design and context of included studies

	Total *N* (%)	
**Study design**		
Cluster RCT	43	53.8%
Individual RCT	37	46.3%
**Country**		
North America	51	63.8%
Europe	23	28.8%
Rest of the world^a^	6	7.5%
**Setting**		
Primary care	72	90.0%
Secondary care inpatient	7	8.8%
Other^b^	1	0.01%
**Health condition/disease of interest**		
Cardiovascular disease	12	15.0%
General health	11	13.8%
Cancer	11	13.8%
Vaccinations	9	11.3%
Mental health	7	8.8%
Diabetes	5	6.3%
Respiratory disease	3	3.8%
Other^c^	22	27.5%

^a^Australia, China, South Africa

^b^Multiple settings (e.g. hospitals + primary healthcare centres)

^c^Obesity, risk factors (e.g. smoking), oral health, sexual health, chronic kidney disease, HIV, pain, neonatal care, orthopaedics

### Characteristics of implementation trials

Of the 80 included studies, 55 (68.8%) were implementation trials, 15 (18.8%) were hybrid type II effectiveness-implementation trials, and 10 (12.5%) were type III hybrid trials. A total of 70.0% (*n* = 56) of included implementation trials tested multicomponent implementation strategies, while 30.0% (*n* = 24) used a single strategy to implement evidence-based interventions. Of those using single implementation strategies, manual or computerised reminders were the most commonly used strategies (66.7%, *n* = 16). Additional file 5 summarises the characteristics of single implementation strategies. Most implementation strategies were compared against usual care or no intervention (75.0%, *n* = 60), but 25.0% were compared with active components such as letters, education, and training. In general, clinical guidelines (22.5%, *n* = 18) were the most frequently implemented evidence-based practice among included trials, followed by disease screening programmes (15.0%, *n* = 12). Seven implementation outcomes (adoption, implementation cost, feasibility, fidelity, penetration, sustainability, acceptability) were mentioned in the included trials, among which adoption/uptake was the most examined (65.0%, *n* = 52), and 12 implementation trials (15.0%) assessed fidelity. See Table 3 for further details.

**Table 3:** Characteristics of trials included in this review

**Type of trials**		
Implementation trials	55	68.8%
Type II hybrid trials	15	18.8%
Type III hybrid trials	10	12.5%
**Implementation strategy**		
Single strategy	24	30.0%
Multicomponent strategy	56	70.0%
**Comparator**		
Usual care/no intervention	60	75.0%
Active comparator^a^	20	25.0%
**Evidence-based intervention**		
Clinical guidelines	18	22.5%
Vaccinations	9	11.3%
Disease screening	12	15.0%
Disease management programme	5	6.3%
Other^b^	36	45.0%
**Implementation outcome**		
Adoption	52	65.0%
Fidelity	12	15.0%
Acceptability/feasibility	3	3.8%
Combinations^c^	13	16.3%

^a^Active implementation components such as letter reminders, educational materials, workshops, questionnaire, training

^b^Evidence-based treatment/service (e.g. breastfeeding, antiplatelet medications, tobacco use prevention and cessation counselling, evidence-based weight management services, advance care planning), model of care, and combinations of screening, vaccinations, and guidelines

^c^Mixed combinations of adoption, implementation cost, feasibility, fidelity, penetration, sustainability, acceptability

### Characteristics of the use of routine data

**Fig. 2:**
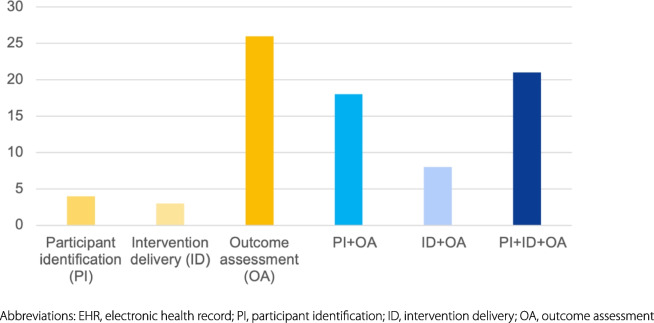
The different tasks for which routine data were used in included trials

**Fig. 3:**
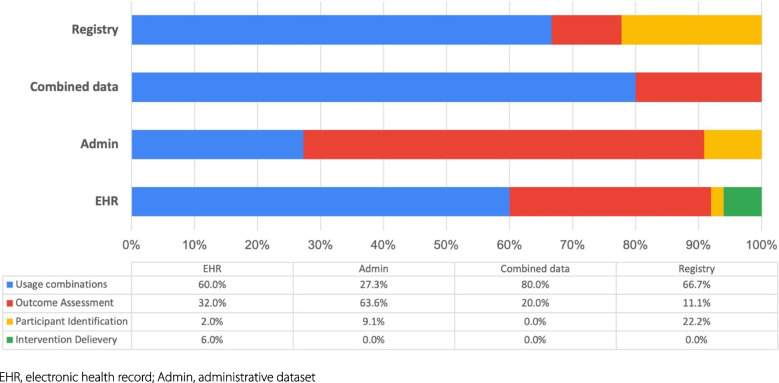
The use of routine data stratified by data types

As shown in Table 4, more than half of implementation trials employed EHR/EMR in the trial execution (62.5%, *n* = 50), while 9 (11.3%) used registry, and 11 (13.8%) used administrative datasets. A total of 61.3% of implementation trials specified the routine healthcare databases used, 16.3% of implementation trials linked data within a single source or across three sources, but fewer than half (*n* = 5, 38.5%) reported methods for data linkage. Figure 2 depicts the use of routine data, solely used for identifying trial participants in 4 (5.0%) studies, for delivering interventions in 3 (3.8%) studies, and for assessing outcomes in 26 (32.5%) studies. The majority of implementation trials (58.8%, *n* = 47) used routine data for multiple purposes. Of the 47 trials, the combination of all three approaches received the most attention (*n* = 21, 44.7%), followed by the combination of participant identification and outcome assessment (*n* = 18, 38.3%). In Fig. 3, among the three types, administrative datasets were mainly used for assessing outcomes, while registries and EHRs were predominantly used in the combination of participant identification, intervention delivery, and/or outcome ascertainment.

**Table 4:** Type and usage of routine data in included trials

Characteristics	*N*	%
Type of routine data		
EHR	50	62.5%
Administrative dataset	11	13.8%
Registry	9	11.3%
Combinations	10	8.0%
Use of routine data		
Participant identification (PI)	4	5.0%
Intervention delivery (ID)	3	3.8%
Outcome assessment (OA)	26	32.5%
Combinations	47	58.8%
PI + OA	18	38.3%
ID + OA	8	17.0%
PI + ID + OA	21	44.7%
Name of databases specified (vs. no/not clear)	49	61.3%
Data linkage (vs. no/not clear)	13	16.3%
Linkage method reported (vs. no/not clear)	5	38.5%

*Abbreviations*: *EHR* Electronic health record, * PI* Participant identification, *ID* Intervention delivery, *OA* Outcome assessment

### Reported rationales, barriers, and facilitators of using routine data in implementation trials

**Fig. 4:**
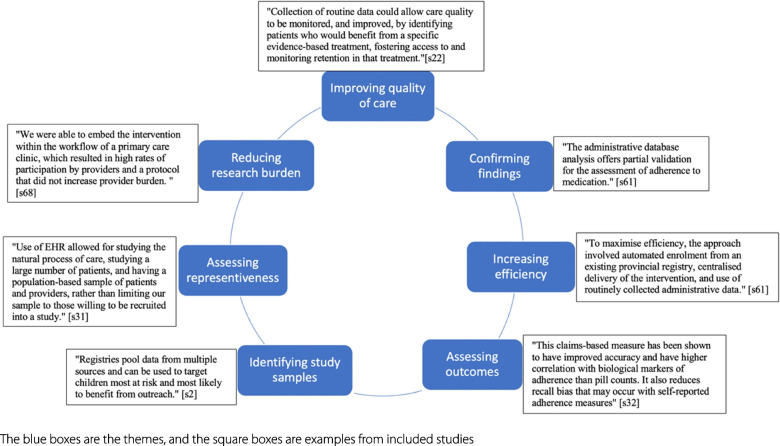
Reported rationales for using routine data in implementation trials

**Fig. 5:**
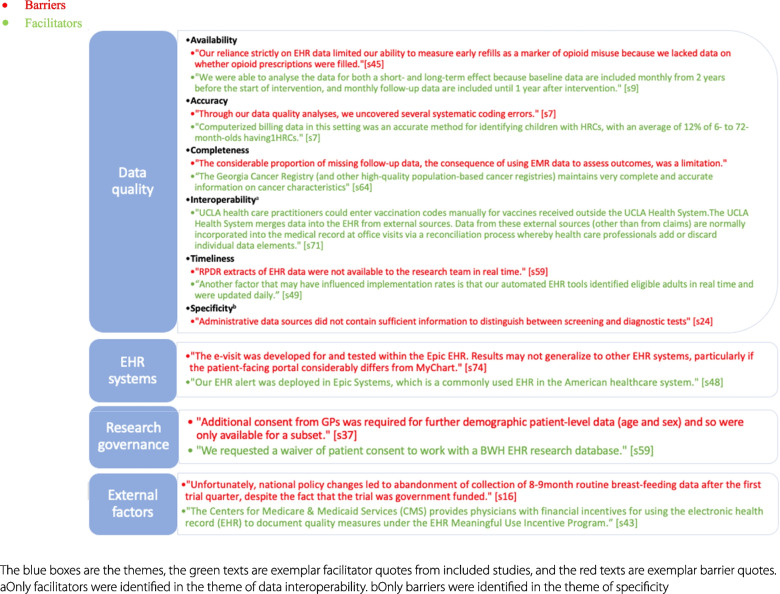
Reported barriers and facilitators

The thematic synthesis used data extracted from 50 studies. Figures 4 and 5 depict all the themes and subthemes identified that capture the reported rationales, facilitators, and barriers, with purposively selected examples. Seven themes capturing the reported rationales were improving quality of care, assessing outcomes, identifying study samples, assessing representativeness, increasing efficiency, confirming findings from other data sources, and reducing the research burden (Fig. 4). Four themes of reported facilitators and barriers of using routine data in implementation trials were data quality (including accuracy, timeliness, availability, interoperability, specificity, completeness), EHR systems (e.g. the choice of EHR vendors), research governance (e.g. informed consent), and external factors such as government policy (Fig. 5). Additional file 6 (themes of rationales) and Additional file 7 (themes of facilitators and barriers) summarise each theme with full examples extracted from included studies. While the theme of EHR systems in Fig. 5 appears to relate directly to the EHR data type, other themes seem not to be associated with particular routine data or implementation trials.

## Discussion

### Summary of findings

This review provides an overview of implementation trials conducted using routine data. We identified 80 trials that evaluated the effect of various implementation strategies designed to implement evidence-based interventions. More than half of implementation trials made use of EHRs in the trial delivery for a combination of participant identification, intervention delivery, and/or outcome ascertainment. Routine data were favoured in assessing implementation outcomes in almost all trials. In addition, we identified rationales for using routine data including improving quality of care, assessing outcomes, identifying study samples, assessing representativeness, increasing efficiency, confirming findings, and reducing the research burden. Data quality, the EHR system, research governance, and external factors such as government policy could act either as facilitators or barriers. Among those 80 trials, the most frequently used implementation strategy was reminders, either manual or computerised. Strategies primarily focused on the adoption/uptake of evidence-based interventions, followed by implementation fidelity.

### Discussion in relation to published literature

#### The use of routine data

We found that most implementation trials chose EHRs as their data sources, which is consistent with the findings from CONSORT-ROUTINE reviews of 263 effectiveness RCTs using registries, administrative databases, and EHR [16–19], in which 70% (*n* = 183) of RCTs used EHRs. Of those 183 trials, 44% used EHR to perform all three functions (i.e. participant identification, intervention delivery, and outcome assessment) [16], which is in line with our findings. EHR contains longitudinal patient medical history which provides rich clinical information such as disease treatment patterns and standards of care [31] and is therefore useful for studying population health. In the context of clinical trials, it is not novel that EHR databases are used for searching patient eligibility; however, this is often done on-site by GPs which is labour intensive [32]. A centralised approach, such as UK Biobank and the Scottish Health Research Register and Biobank, provides in-depth biological and medical data for screening and locating potentially eligible trial participants, which can reduce the research burden for study personnel and facilitate involvement of patients [33]. The use of EHR data to enhance recruitment has previously been endorsed by the PRINCIPLE trial [34], a UK-wide clinical study investigating potential treatments for COVID-19 in the community. The participants joined the trial online, and their eligibility was checked centrally via data received from GP records. This centralised approach was associated with increased recruitment of people with positive COVID-19 test results, demonstrating the value of routine data in reaching out to a wider population.

The capability of routine data in providing outcome assessment with potentially readily available data is also appealing. A search of NIHR HTA-funded trials registered in 2019 found that 47% of the trials planned to use routine data as a source of outcome data [35]. In our review of implementation trials, nearly all of the trials (91.3%) used routine data for outcome ascertainment. This allows outcomes in the whole eligible population to be measured (as opposed to outcomes in trial participants) and is practical because outcomes in implementation trials are typically adoption rates or service utility, which are routinely recorded for whole populations. In contrast, in the context of effectiveness RCTs, some common outcomes of interest such as biomarkers and patient-reported outcomes are not routinely collected in the databases [11]; thus, other data collection methods may be needed.

#### Rationales, facilitators, and barriers

EHRs, administrative databases, and registries have been promoted for over a decade as offering opportunities for supporting the design and execution of clinical trials [29, 36]. In 2018, a UK National Workshop was held by the National Institute of Health Research (NIHR), Health Data Research UK, and Clinical Practice Research Datalink, to promote the agenda for data-enabled clinical trials [34]. The COVID-19 pandemic has further accelerated the potential of this innovative trial design. For example, RECOVERY, the world’s largest clinical trial evaluating the potential treatments for COVID-19, has endorsed the vital role of routine data in its success in finding effective treatments [37].

Our findings on the challenges and facilitators of using routine data resonate with the findings in studies of effectiveness RCTs [11, 38–40]. In addition to the rationales identified in our review, the ability to provide more generalisable outcomes and conduct long-term follow-up seems to attract the most attention [11, 39, 40]. For example, a 9-year follow-up of the ASCEND trial evaluating the effect of aspirin on cardiovascular events in patients with diabetes is supported by linking electronic hospital episode data [41]. This is also of direct relevance to implementation trials, where the ultimate goal is to implement, scale up, and sustain evidence-based innovations in routine practices, which requires substantial time and resource commitment. With the help of routine data, long-term benefits and harms can be monitored among general populations for extended periods after the trial termination [12].

Nevertheless, obstacles exist that may prevent routine data from achieving its full potential. Data quality is the main concern and has been extensively discussed in healthcare data research. In this review, one trial criticised the EHR data extracted for the research team because they were not in real time [s59], which largely impeded data analysis. Timeliness is a key measurement of data quality; in implementation trials, it is also an enabler of seamlessly translating research findings into daily practice. While some routine data such as EHRs are updated regularly, others such as hospital episode statistics are periodically updated which may not support real-time trial delivery.

Since a single data source may not contain all the information required by the trial, trialists may use and link multiple data sources to evaluate all outcomes [32]. The use of data linkage offers opportunities to address the representativeness of the samples and assess the generalisability of the results, as endorsed by the included studies [s31, s61]. No barriers to data interoperability were noted in the trials collated in this review, though only 13 trials (16.3%) performed and reported data linkage. Nonetheless, the concerns with linking routine data have been widely acknowledged in the literature, for example errors in data linkage (e.g. missed matches or false matches) leading to inaccurate results [42], and technical challenges (e.g. handling changing data), the sheer volume of datasets and different linkage scenarios, increase the complexity and the costs [43]. Data quality needs to be reasonably assured in the trial context especially when it relates to outcome measurement, since poor data quality may undermine trial findings. Aside from data quality issues, common barriers such as costs and training associated with obtaining and managing data [11], complex and time-consuming research governance, and regulatory approvals [11, 38, 40] have been identified in previous studies. Addressing these barriers is of utmost importance to improve trial quality and efficiency.

#### Randomised implementation trials

In this review, identifying randomised implementation trials was a challenge, despite guidelines on planning and reporting implementation studies [1, 30]. Issues, such as lacking implementation trial labels in trial papers, not indicating implementation research in titles and abstracts, not explicitly naming implementation strategies and implementation outcomes, and confusing terminologies occurring in different publications, led to poor reporting of implementation trials. Similar results have been found in other reviews of implementation studies [44, 45].

Standardising and harmonising the reporting of implementation trials will ease the replication of effective strategies for improving implementation outcomes, thus enhancing the integration of implementation science discoveries into routine practice [46]. Furthermore, it will increase the visibility of literature, thereby improving the quality of evidence synthesis in systematic reviews of implementation trials. Informed by guidelines for designing [1] and reporting [30] implementation studies, we have summarised some key considerations in reporting implementation trials in Table 5. Additionally, the current definition of an implementation trial seems insufficient to capture the full characteristics, given the wide variation in designing and reporting, as well as the introduction of hybrid designs. Therefore, we clarified the definition of an implementation trial as a “research design assessing the effects of implementation strategy(ies) on promoting the evidence-based practice into the daily practice, with the primary focus on the implementation outcome that is distinct from service and patient outcomes”.

**Table 5:** Summary of recommendations

Implementation strategy	1) Using the convention of distinguishing the implementation strategy (the initiative(s) being tested in an implementation trial) from the evidence-based intervention that is being implemented [29] 2) Describing the common implementation strategies according to an existing standardisation or taxonomy [5] 3) Specifying the unique strategies by (1) the actor, (2) the action, (3) action target, (4) temporality (i.e. timing and sequencing), (5) dose, (6) implementation outcomes affected, and (7) theoretical, empirical, or pragmatic justification [5]
Implementation outcome	1) Distinguish implementation outcomes from clinical outcomes and set priority for implementation outcomes 2) Employ standardised language in labelling and describing the implementation outcomes, where applicable

### Implication: realising the potential of routine data in implementation trials

The use of routine data in RCTs has been widely advocated, but this is particularly pertinent in implementation trials. Unlike the evaluation of healthcare interventions on individual health outcomes, implementation research concerns outcome improvements at the whole population level [5]. While routinely collected data capture information in large populations, it provides more comprehensive indicators for evaluating interventions that are directed to the whole population compared to sample-based data sets [47]. They are therefore crucial in implementation research to facilitate translation of research findings into practice.

Implementation science acts proactively to bridge the gap between research evidence and routine healthcare, an implementation trial tests strategies to implement evidence-based clinical innovations into wider practice [48]. Data derived from routine healthcare is an essential connector that can be fed into implementation trials to close the loop of research-practice translation to achieve a continuous optimisation of healthcare interventions and their integration into the real world. This is also an illustration of the learning healthcare system, where knowledge generation is embedded in daily practice for continuous improvement and innovation [49]. Chambers and colleagues [50] addressed the value of implementation science in learning systems and summarised one of the potential synergies between the two domains as “support for implementation of effective practices”. Indeed, by harnessing the power of routine data, implementation science could maximise the capability of closing the known gap, thereby bringing mutual benefit to both scientific research and healthcare routine practice and improving the impact on whole population health.

### Strengths and limitations

A key strength of this review is the novelty of the topic, in that no such review has been done in this emerging field. Although we employed a systematic approach to identifying and summarising implementation trials, several limitations require consideration.

Firstly, although the original review protocol focused on the use of routine data in implementation trials, the final review also reported the characteristics of implementation trial designs. We believe this provides valuable context and adds breadth to our research and will be of use to readers. Secondly, our tailored search strategy may have restricted the number of trials identified and led to the underrepresentation of relevant studies not explicit in their reporting of routine data use, possibly affecting the breadth of our findings. This may also introduce a bias towards trials with more meticulous reporting, or in which routine data had a prominent role (e.g. as a primary outcome measure), and potentially overlooking trials where the routine data usage was not featured in the abstract (e.g. for identifying suitable sites). Thirdly, our study did not specifically seek out process evaluations published as separate papers, so we will have overlooked trials in which the routine data contributed exclusively to process outcomes. Future research should include process evaluations to obtain insights on the barriers and facilitators related to the use of routine data, providing a more comprehensive understanding. A further limitation is that we have not summarised all the methodological characteristics of implementation trials using routine data, so may have neglected other potential key characteristics. For example, items listed in CONSORT-ROUTINE checklist [10], such as “information on how to access the list of codes and algorithms used to define or derive the outcomes from the cohort or routinely collected database(s) used to conduct the trial, information on accuracy and completeness of outcome variables, and methods used to validate accuracy and completeness”, are important factors to consider in trials conducted using routine data. In addition, we excluded non-English studies and long-term trial follow-up reports, which could have contributed to the findings. Finally, this review is constrained by the absence of a list of all excluded reports due to a system crash, no complete duplicate study selection and extraction and the lack of quality assessment for the included trials, potentially affecting the results’ reliability and generalisability.

## Conclusion

There is a need to enhance adherence to guidance on designing and reporting implementation studies and specifically to harmonise the language used in describing implementation strategies and implementation outcomes. Routine healthcare data offer promise in supporting the implementation of evidence-based interventions and are frequently employed in assessing implementation outcomes. EHRs are widely used in terms of participant identification, outcome ascertainment and intervention delivery. In the meantime, researchers should familiarise themselves with the barriers and facilitators to using routine data, and efforts could be made to improve data quality to overcome some barriers.

### Supplementary Information

**Additional file 1: Additional file 1.** PRISMA 2020 Checklist. **Additional file 2.** Search Strategy. **Additional file 3.** Description of implementation outcomes according to Proctor et al., 2010. **Additional file 4.** References for included trials. **Additional file 5.** Summary of single implementation strategies used in included trials. **Additional file 6.** Reported rationale themes with full examples. **Additional file 7.** Reported facilitators and barriers with full examples.

## Data Availability

All data cited in this review derives from published papers and are therefore already available.

## Abbreviations

EHR: Electronic health record
RCT: Randomised controlled trial
CONSORT-ROUTINE: Consolidated Standards of Reporting Trials extension for trials conducted using cohorts and routinely collected data
PI: Participant identification
ID: Intervention delivery
OA: Outcome assessment
