# Supplementary material for: Use of routine healthcare data in randomised implementation trials: a methodological mixed-methods systematic review
Source: Implement Sci. 2023 Oct 2;18:47. doi: 10.1186/s13012-023-01300-4 (PMC10544368; doi:10.1186/s13012-023-01300-4)

Additional file 1. PRISMA 2020 Checklist

Section and Topic	Item #	Checklist item	Location where item is reported
**TITLE**			
Title	1	Identify the report as a systematic review.	Title
**ABSTRACT**			
Abstract	2	See the PRISMA 2020 for Abstracts checklist.	Abstract
**INTRODUCTION**			
Rationale	3	Describe the rationale for the review in the context of existing knowledge.	Background, paragraph 1-2
Objectives	4	Provide an explicit statement of the objective(s) or question(s) the review addresses.	Background, paragraph 3
**METHODS**			
Eligibility criteria	5	Specify the inclusion and exclusion criteria for the review and how studies were grouped for the syntheses.	Definitions, inclusion and exclusion criteria, table 1
Information sources	6	Specify all databases, registers, websites, organisations, reference lists and other sources searched or consulted to identify studies. Specify the date when each source was last searched or consulted.	Systematic searches
Search strategy	7	Present the full search strategies for all databases, registers and websites, including any filters and limits used.	Additional file 2
Selection process	8	Specify the methods used to decide whether a study met the inclusion criteria of the review, including how many reviewers screened each record and each report retrieved, whether they worked independently, and if applicable, details of automation tools used in the process.	Data screening
Data collection process	9	Specify the methods used to collect data from reports, including how many reviewers collected data from each report, whether they worked independently, any processes for obtaining or confirming data from study investigators, and if applicable, details of automation tools used in the process.	Data extraction and analysis
Data items	10a	List and define all outcomes for which data were sought. Specify whether all results that were compatible with each outcome domain in each study were sought (e.g. for all measures, time points, analyses), and if not, the methods used to decide which results to collect.	Data extraction and analysis
	10b	List and define all other variables for which data were sought (e.g. participant and intervention characteristics, funding sources). Describe any assumptions made about any missing or unclear information.	Data extraction and analysis, table 1
Study risk of bias assessment	11	Specify the methods used to assess risk of bias in the included studies, including details of the tool(s) used, how many reviewers assessed each study and whether they worked independently, and if applicable, details of automation tools used in the process.	N/A
Effect measures	12	Specify for each outcome the effect measure(s) (e.g. risk ratio, mean difference) used in the synthesis or presentation of results.	N/A, Narrative synthesis
Synthesis methods	13a	Describe the processes used to decide which studies were eligible for each synthesis (e.g. tabulating the study intervention characteristics and comparing against the planned groups for each synthesis (item #5)).	Mixed method analysis
	13b	Describe any methods required to prepare the data for presentation or synthesis, such as handling of missing summary statistics, or data conversions.	Analysis and synthesis of

Additional file 1_PRISMA 2020 Checklist

Section and Topic	Item #	Checklist item	Location where item is reported
			findings
	13c	Describe any methods used to tabulate or visually display results of individual studies and syntheses.	Descriptive tables/figures
	13d	Describe any methods used to synthesize results and provide a rationale for the choice(s). If meta-analysis was performed, describe the model(s), method(s) to identify the presence and extent of statistical heterogeneity, and software package(s) used.	Data extraction and analysis
	13e	Describe any methods used to explore possible causes of heterogeneity among study results (e.g. subgroup analysis, meta-regression).	N/A, Narrative synthesis
	13f	Describe any sensitivity analyses conducted to assess robustness of the synthesized results.	N/A
Reporting bias assessment	14	Describe any methods used to assess risk of bias due to missing results in a synthesis (arising from reporting biases).	N/A
Certainty assessment	15	Describe any methods used to assess certainty (or confidence) in the body of evidence for an outcome.	N/A
**RESULTS**			
Study selection	16a	Describe the results of the search and selection process, from the number of records identified in the search to the number of studies included in the review, ideally using a flow diagram.	Results section; PRISMA flow diagram
	16b	Cite studies that might appear to meet the inclusion criteria, but which were excluded, and explain why they were excluded.	PRISMA flow diagram, additional file 2
Study characteristics	17	Cite each included study and present its characteristics.	Study characteristics, Table 2,3,4, Figure 2,3,4,5
Risk of bias in studies	18	Present assessments of risk of bias for each included study.	N/A
Results of individual studies	19	For all outcomes, present, for each study: (a) summary statistics for each group (where appropriate) and (b) an effect estimate and its precision (e.g. confidence/credible interval), ideally using structured tables or plots.	Descriptive statistics and narrative synthesis, Table 2,3,4, Figure 2,3,4,5
Results of syntheses	20a	For each synthesis, briefly summarise the characteristics and risk of bias among contributing studies.	Study characteristics sections

Additional file 1_PRISMA 2020 Checklist

Section and Topic	Item #	Checklist item	Location where item is reported
	20b	Present results of all statistical syntheses conducted. If meta-analysis was done, present for each the summary estimate and its precision (e.g. confidence/credible interval) and measures of statistical heterogeneity. If comparing groups, describe the direction of the effect.	N/A
	20c	Present results of all investigations of possible causes of heterogeneity among study results.	N/A
	20d	Present results of all sensitivity analyses conducted to assess the robustness of the synthesized results.	N/A
Reporting biases	21	Present assessments of risk of bias due to missing results (arising from reporting biases) for each synthesis assessed.	N/A
Certainty of evidence	22	Present assessments of certainty (or confidence) in the body of evidence for each outcome assessed.	N/A, this is a review of methodology, not a review of findings
**DISCUSSION**			
Discussion	23a	Provide a general interpretation of the results in the context of other evidence.	Summary of findings
	23b	Discuss any limitations of the evidence included in the review.	Strength and limitations
	23c	Discuss any limitations of the review processes used.	Strength and limitations
	23d	Discuss implications of the results for practice, policy, and future research.	Implications
**OTHER INFORMATION**			
Registration and protocol	24a	Provide registration information for the review, including register name and registration number, or state that the review was not registered.	Abstract
	24b	Indicate where the review protocol can be accessed, or state that a protocol was not prepared.	Abstract
	24c	Describe and explain any amendments to information provided at registration or in the protocol.	N/A
Support	25	Describe sources of financial or non-financial support for the review, and the role of the funders or sponsors in the review.	Declarations
Competing interests	26	Declare any competing interests of review authors.	Declarations
Availability of data, code and other materials	27	Report which of the following are publicly available and where they can be found: template data collection forms; data extracted from included studies; data used for all analyses; analytic code; any other materials used in the review.	Declarations

From: Page MJ, McKenzie JE, Bossuyt PM, Boutron I, Hoffmann TC, Mulrow CD, et al. The PRISMA 2020 statement: an updated guideline for reporting systematic reviews. BMJ 2021;372:n71. doi: 10.1136/bmj.n71

For more information, visit: http://www.prisma-statement.org/

Additional file 2. Search Strategy

Ovid MEDLINE(R) and Epub Ahead of Print, In-Process, In-Data-Review & Other Non-Indexed Citations, Daily and Versions(R) <1946 to November 17, 2021>

1	"routine health data*".ti,ab,kw.	105
2	(routinely collected adj5 data*).ti,ab,kw.	4120
3	(((healthcare adj3 data*) or health) adj3 data*).ti,ab,kw.	67403
4	exp Electronic Health Records/	24454
5	exp Health records, Personal/	2305
6	exp Medical Records Systems, Computerized/	44982
7	administrative data*.ab,ti,kf. or (administrative adj5 data*).ti,ab,kw.	21725
8	"National Program of Cancer Registries"/	6
9	Registries/	100425
10	(routine adj5 data*).ti,ab,kw.	10955
11	1 or 2 or 3 or 4 or 5 or 6 or 7 or 8 or 9 or 10	235176
12	trial.ti,ab,kw.	670298
13	(implement* or effectiveness or efficacy or real-world or "real world" or pragmatic or evidence-based or "evidence based" or real-life or "real life" or hybrid).ti,ab,kw.	2181529
14	12 and 13	220814
15	11 and 14	3997

Cochrane Library
Date of search: 17/11/2021
Results: 462 trials

ID	Search
#1	MeSH descriptor: [Randomized Controlled Trials as Topic] explode all trees
#2	MeSH descriptor: [Pragmatic Clinical Trials as Topic] explode all trees
#3	(trial):ti,ab,kw
#4	MeSH descriptor: [Implementation Science] explode all trees
#5	#1 OR #2 OR #3
#6	(implement*):ti,ab,kw
#7	#4 OR #6
#8	#5 AND #7
#9	MeSH descriptor: [Routinely Collected Health Data] explode all trees
#10	MeSH descriptor: [Electronic Health Records] explode all trees
#11	MeSH descriptor: [Health Records, Personal] explode all trees
#12	(administrative data*):ti,ab,kw
#13	MeSH descriptor: [Internet of Things] explode all trees
#14	MeSH descriptor: [Registries] explode all trees
#15	(hospital episode data*):ti,ab,kw
#16	#9 OR #10 OR #11 OR #12 OR #13 OR #14 OR #15
#17	#8 AND #16 with Publication Year from 2000 to 2021, in Trials

Additional file 3. Description of implementation outcomes according to Proctor et al., 2010

IMPLEMENTATION OUTCOME	MAY ALSO REFER TO	DEFINITION
**ACCEPTABILITY**	Satisfaction	*The perception among implementation stakeholders that a given treatment, service, practice, or innovation is agreeable, palatable, or satisfactory.*
**ADOPTION**	Uptake; Utilization; Intention to try	*The intention, initial decision, or action to try or employ an innovation or evidence-based practice.*
**APPROPRIATENESS**	Perceived fit; Relevance; Usefulness;	*The perceived fit, relevance, or compatibility of the innovation or evidence-based practice for a given practice setting, provider, or consumer; and/or perceived fit of the innovation to address a particular issue or problem.*
**FEASIBILITY**	Actual fit; Utility; Practicability	*The extent to which a new treatment, or an innovation, can be successfully used or carried out within a given agency or setting.*
**FIDELITY**	Adherence; Delivered as intended; Quality of program delivery	*The degree to which an intervention was implemented as it was prescribed in the original protocol or as it was intended by the program developers*
**IMPLEMENTATION COST**	Cost-effectiveness; Cost-benefit	*The cost impact of an implementation effort.*
**PENETRATION**	Spread	*The integration of a practice within a service setting and its subsystems.*
**SUSTAINABILITY**	Maintenance; Continuation; Incorporation;	*The extent to which a newly implemented treatment is maintained or institutionalized within a service setting's ongoing, stable operations.*

Proctor E, Silmere H, Raghavan R, Hovmand P, Aarons G, Bunger A, Griffey R, Hensley M. Outcomes for implementation research: conceptual distinctions, measurement challenges, and research agenda. Administration and policy in mental health and mental health services research. 2011 Mar;38(2):65-76.

Additional file 4. References for included trials

s1. Barr JK, Franks AL, Lee NC, et al. A randomized intervention to improve ongoing participation in mammography. Am J Manag Care 2001;7(9):887-94.
s2. Wilcox SA, Koepke CP, Levenson R, et al. Registry-driven, community-based immunization outreach: a randomized controlled trial. Am J Public Health 2001;91(9):1507-11.
s3. Newell SA, Sanson-Fisher RW, Girgis A, et al. Can personal health record booklets improve cancer screening behaviors? Am J Prev Med 2002;22(1):15-22.
s4. Stroebel RJ, Scheitel SM, Fitz JS, et al. A Randomized Trial of Three Diabetes Registry Implementation Strategies in a Community Internal Medicine Practice. The Joint Commission Journal on Quality Improvement 2002;28(8):441-50.
s5. Tierney WM, Overhage JM, Murray MD, et al. Effects of computerized guidelines for managing heart disease in primary care. J Gen Intern Med 2003;18(12):967-76.
s6. Bahrami M, Deery C, Clarkson JE, et al. Effectiveness of strategies to disseminate and implement clinical guidelines for the management of impacted and unerupted third molars in primary dental care, a cluster randomised controlled trial. Br Dent J 2004;197(11):691-6; discussion 88.
s7. Daley MF, Barrow J, Pearson K, et al. Identification and recall of children with chronic medical conditions for influenza vaccination. Pediatrics 2004;113(1 Pt 1):e26-33.
s8. Subramanian U, Fihn SD, Weinberger M, et al. A controlled trial of including symptom data in computer-based care suggestions for managing patients with chronic heart failure. Am J Med 2004;116(6):375-84.
s9. Witt K, Knudsen E, Ditlevsen S, et al. Academic detailing has no effect on prescribing of asthma medication in Danish general practice: a 3-year randomized controlled trial with 12-monthly follow-ups. Fam Pract 2004;21(3):248-53.
s10. Kempe A, Daley MF, Barrow J, et al. Implementation of universal influenza immunization recommendations for healthy young children: results of a randomized, controlled trial with registry-based recall. Pediatrics 2005;115(1):146-54.
s11. Stein K, Lewendon G, Jenkins R, et al. Improving uptake of cervical cancer screening in women with prolonged history of non-attendance for screening: a randomized trial of enhanced invitation methods. J Med Screen 2005;12(4):185-9.
s12. Cleland JA, Hall S, Price D, et al. An exploratory, pragmatic, cluster randomised trial of practice nurse training in the use of asthma action plans. Prim Care Respir J 2007;16(5):311-8.
s13. Harari D, Iliffe S, Kharicha K, et al. Promotion of health in older people: a randomised controlled trial of health risk appraisal in British general practice. Age Ageing 2008;37(5):565-71.
s14. Wright A, Poon EG, Wald J, et al. Effectiveness of health maintenance reminders provided directly to patients. AMIA Annu Symp Proc 2008:1183.
s15. Yano EM, Rubenstein LV, Farmer MM, et al. Targeting primary care referrals to smoking cessation clinics does not improve quit rates: implementing evidence-based interventions into practice. Health Serv Res 2008;43(5 Pt 1):1637-61.
s16. Hoddinott P, Britten J, Prescott GJ, et al. Effectiveness of policy to provide breastfeeding groups (BIG) for pregnant and breastfeeding mothers in primary care: cluster randomised controlled trial. BMJ 2009;338:a3026.
s17. Linder JA, Rigotti NA, Schneider LI, et al. An electronic health record-based intervention to improve tobacco treatment in primary care: a cluster-randomized controlled trial. Arch Intern Med 2009;169(8):781-7.

s18. Peremans L, Rethans JJ, Verhoeven V, et al. Empowering patients or general practitioners? A randomised clinical trial to improve quality in reproductive health care in Belgium. Eur J Contracept Reprod Health Care 2010;15(4):280-9.
s19. Chaney EF, Rubenstein LV, Liu CF, et al. Implementing collaborative care for depression treatment in primary care: a cluster randomized evaluation of a quality improvement practice redesign. Implement Sci 2011;6:121.
s20. Krist AH, Woolf SH, Rothemich SF, et al. Interactive preventive health record to enhance delivery of recommended care: a randomized trial. Ann Fam Med 2012;10(4):312-9.
s21. Amemori M, Virtanen J, Korhonen T, et al. Impact of educational intervention on implementation of tobacco counselling among oral health professionals: a cluster-randomized community trial. Community Dent Oral Epidemiol 2013;41(2):120-9.
s22. Cohen AN, Chinman MJ, Hamilton AB, et al. Using patient-facing kiosks to support quality improvement at mental health clinics. Med Care 2013;51(3 Suppl 1):S13-20.
s23. French SD, McKenzie JE, O'Connor DA, et al. Evaluation of a theory-informed implementation intervention for the management of acute low back pain in general medical practice: the IMPLEMENT cluster randomised trial. PLoS One 2013;8(6):e65471.
s24. Green BB, Wang CY, Anderson ML, et al. An automated intervention with stepped increases in support to increase uptake of colorectal cancer screening: a randomized trial. Ann Intern Med 2013;158(5 Pt 1):301-11.
s25. Kilgore ML, Outman R, Locher JL, et al. Multimodal intervention to improve osteoporosis care in home health settings: results from a cluster randomized trial. Osteoporos Int 2013;24(10):2555-60.
s26. Persell SD, Lloyd-Jones DM, Friesema EM, et al. Electronic health record-based patient identification and individualized mailed outreach for primary cardiovascular disease prevention: a cluster randomized trial. J Gen Intern Med 2013;28(4):554-60.
s27. Smidth M, Christensen MB, Fenger-Gron M, et al. The effect of an active implementation of a disease management programme for chronic obstructive pulmonary disease on healthcare utilization--a cluster-randomised controlled trial. BMC Health Serv Res 2013;13:385.
s28. Dombkowski KJ, Costello LE, Harrington LB, et al. Age-specific strategies for immunization reminders and recalls: a registry-based randomized trial. Am J Prev Med 2014;47(1):1-8.
s29. Radde K, Gottschalk A, Bussas U, et al. Invitation to cervical cancer screening does increase participation in Germany: Results from the MARZY study. Int J Cancer 2016;139(5):1018-30.
s30. Taveras EM, Marshall R, Horan CM, et al. Improving children's obesity-related health care quality: process outcomes of a cluster-randomized controlled trial. Obesity (Silver Spring) 2014;22(1):27-31.
s31. Mertens JR, Chi FW, Weisner CM, et al. Physician versus non-physician delivery of alcohol screening, brief intervention and referral to treatment in adult primary care: the ADVISe cluster randomized controlled implementation trial. Addict Sci Clin Pract 2015;10:26.
s32. Palacio AM, Uribe C, Hazel-Fernandez L, et al. Can phone-based motivational interviewing improve medication adherence to antiplatelet medications after a coronary stent among racial minorities? A randomized trial. J Gen Intern Med 2015;30(4):469-75.
s33. Sinnema H, Majo MC, Volker D, et al. Effectiveness of a tailored implementation programme to improve recognition, diagnosis and treatment of anxiety and depression in general practice: a cluster randomised controlled trial. Implement Sci 2015;10:33.

s34. Forberg U, Unbeck M, Wallin L, et al. Effects of computer reminders on complications of peripheral venous catheters and nurses' adherence to a guideline in paediatric care--a cluster randomised study. Implement Sci 2016;11:10.
s35. Guiriguet C, Munoz-Ortiz L, Buron A, et al. Alerts in electronic medical records to promote a colorectal cancer screening programme: a cluster randomised controlled trial in primary care. Br J Gen Pract 2016;66(648):e483-90.
s36. Hemming K, Ryan R, Gill P, et al. Targeted case finding in the prevention of cardiovascular disease: a stepped wedge cluster randomised controlled trial. Br J Gen Pract 2016;66(651):e758-67.
s37. Herrett E, Williamson E, van Staa T, et al. Text messaging reminders for influenza vaccine in primary care: a cluster randomised controlled trial (TXT4FLUJAB). BMJ Open 2016;6(2):e010069.
s38. Lin CJ, Nowalk MP, Pavlik VN, et al. Using the 4 pillars practice transformation program to increase adult influenza vaccination and reduce missed opportunities in a randomized cluster trial. BMC Infect Dis 2016;16(1):623.
s39. McDermott L, Wright AJ, Cornelius V, et al. Enhanced invitation methods and uptake of health checks in primary care: randomised controlled trial and cohort study using electronic health records. Health Technol Assess 2016;20(84):1-92.
s40. Nowalk MP, Lin CJ, Pavlik VN, et al. Using the 4 Pillars Practice Transformation Program to increase adult Tdap immunization in a randomized controlled cluster trial. Vaccine 2016;34(41):5026-33.
s41. Arts DL, Abu-Hanna A, Medlock SK, et al. Effectiveness and usage of a decision support system to improve stroke prevention in general practice: A cluster randomized controlled trial. PLoS One 2017;12(2):e0170974.
s42. Chinman M, McCarthy S, Hannah G, et al. Using Getting To Outcomes to facilitate the use of an evidence-based practice in VA homeless programs: a cluster-randomized trial of an implementation support strategy. Implement Sci 2017;12(1):34.
s43. Fitzpatrick SL, Dickins K, Avery E, et al. Effect of an obesity best practice alert on physician documentation and referral practices. Transl Behav Med 2017;7(4):881-90.
s44. Hannon TS, Dugan TM, Saha CK, et al. Effectiveness of Computer Automation for the Diagnosis and Management of Childhood Type 2 Diabetes: A Randomized Clinical Trial. JAMA Pediatr 2017;171(4):327-34.
s45. Liebschutz JM, Xuan Z, Shanahan CW, et al. Improving Adherence to Long-term Opioid Therapy Guidelines to Reduce Opioid Misuse in Primary Care: A Cluster-Randomized Clinical Trial. JAMA Intern Med 2017;177(9):1265-72.
s46. Watkins KE, Ober AJ, Lamp K, et al. Collaborative Care for Opioid and Alcohol Use Disorders in Primary Care: The SUMMIT Randomized Clinical Trial. JAMA Intern Med 2017;177(10):1480-88.
s47. Bruyndonckx R, Verhoeven V, Anthierens S, et al. The implementation of academic detailing and its effectiveness on appropriate prescribing of pain relief medication: a real-world cluster randomized trial in Belgian general practices. Implement Sci 2018;13(1):6.
s48. Chak E, Taefi A, Li CS, et al. Electronic Medical Alerts Increase Screening for Chronic Hepatitis B: A Randomized, Double-Blind, Controlled Trial. Cancer Epidemiol Biomarkers Prev 2018;27(11):1352-57.
s49. Coronado GD, Petrik AF, Vollmer WM, et al. Effectiveness of a Mailed Colorectal Cancer Screening Outreach Program in Community Health Clinics: The STOP CRC Cluster Randomized Clinical Trial. JAMA Intern Med 2018;178(9):1174-81.
s50. Drake C, Meade C, Hull SK, et al. Integration of Personalized Health Planning and Shared Medical Appointments for Patients with Type 2 Diabetes Mellitus. South Med J 2018;111(11):674-82.

s51. McClure JB, Anderson ML. Evaluation of a population-level strategy to promote tobacco treatment use among insured smokers: a pragmatic, randomized trial. BMC Public Health 2018;18(1):228.
s52. Nielson CM, Rivelli JS, Fuoco MJ, et al. Effectiveness of automated and live phone reminders after mailed-FIT outreach in a pilot randomized trial. Prev Med Rep 2018;12:210-13.
s53. Pallan M, Hurley KL, Griffin T, et al. A cluster-randomised feasibility trial of a children's weight management programme: the Child weigHt mANaGement for Ethnically diverse communities (CHANGE) study. Pilot Feasibility Stud 2018;4:175.
s54. Relton C, Strong M, Thomas KJ, et al. Effect of Financial Incentives on Breastfeeding: A Cluster Randomized Clinical Trial. JAMA Pediatr 2018;172(2):e174523.
s55. Svane JK, Chiou ST, Groene O, et al. A WHO-HPH operational program versus usual routines for implementing clinical health promotion: an RCT in health promoting hospitals (HPH). Implement Sci 2018;13(1):153.
s56. Bauer MS, Miller CJ, Kim B, et al. Effectiveness of Implementing a Collaborative Chronic Care Model for Clinician Teams on Patient Outcomes and Health Status in Mental Health: A Randomized Clinical Trial. JAMA Netw Open 2019;2(3):e190230.
s57. Gold R, Bunce A, Cowburn S, et al. Does increased implementation support improve community clinics' guideline-concordant care? Results of a mixed methods, pragmatic comparative effectiveness trial. Implement Sci 2019;14(1):100.
s58. King C, Llewellyn C, Shahmanesh M, et al. Sexual risk reduction interventions for patients attending sexual health clinics: a mixed-methods feasibility study. Health Technol Assess 2019;23(12):1-122.
s59. Wang SV, Rogers JR, Jin Y, et al. Stepped-wedge randomised trial to evaluate population health intervention designed to increase appropriate anticoagulation in patients with atrial fibrillation. BMJ Qual Saf 2019;28(10):835-42.
s60. Cai Y, Gong W, He H, et al. Mobile Texting and Lay Health Supporters to Improve Schizophrenia Care in a Resource-Poor Community in Rural China (LEAN Trial): Randomized Controlled Trial Extended Implementation. J Med Internet Res 2020;22(12):e22631.
s61. Ivers NM, Schwalm JD, Bouck Z, et al. Interventions supporting long term adherence and decreasing cardiovascular events after myocardial infarction (ISLAND): pragmatic randomised controlled trial. BMJ 2020;369:m1731.
s62. Japuntich SJ, Hammett PJ, Rogers ES, et al. Effectiveness of Proactive Tobacco Cessation Treatment Outreach Among Smokers With Serious Mental Illness. Nicotine Tob Res 2020;22(9):1433-38.
s63. Kapoor A, Amroze A, Vakil F, et al. SUPPORT-AF II: Supporting Use of Anticoagulants Through Provider Profiling of Oral Anticoagulant Therapy for Atrial Fibrillation: A Cluster-Randomized Study of Electronic Profiling and Messaging Combined With Academic Detailing for Providers Making Decisions About Anticoagulation in Patients With Atrial Fibrillation. Circ Cardiovasc Qual Outcomes 2020;13(2):e005871.
s64. Lipscomb J, Escoffery C, Gillespie TW, et al. Improving Screening Uptake among Breast Cancer Survivors and Their First-Degree Relatives at Elevated Risk to Breast Cancer: Results and Implications of a Randomized Study in the State of Georgia. Int J Environ Res Public Health 2020;17(3).
s65. Lum HD, Dukes J, Daddato AE, et al. Effectiveness of Advance Care Planning Group Visits Among Older Adults in Primary Care. J Am Geriatr Soc 2020;68(10):2382-89.
s66. Lowery JT, Horick N, Kinney AY, et al. A randomized trial to increase colonoscopy screening in members of high-risk families in the colorectal cancer family registry and cancer genetics network. Cancer Epidemiol Biomarkers Prev 2014;23(4):601-10.

s67. Moen KA, Kumar B, Igland J, et al. Effect of an Intervention in General Practice to Increase the Participation of Immigrants in Cervical Cancer Screening: A Cluster Randomized Clinical Trial. JAMA Netw Open 2020;3(4):e201903.
s68. Peralta CA, Frigaard M, Rolon L, et al. Screening for CKD To Improve Processes of Care among Nondiabetic Veterans with Hypertension: A Pragmatic Cluster-Randomized Trial. Clin J Am Soc Nephrol 2020;15(2):174-81.
s69. Sabo RT, Etz RS, Gonzalez MM, et al. Low-Intensity Intervention Supports Diabetes Registry Implementation: A Cluster-Randomized Trial in the Ambulatory Care Outcomes Research Network (ACORN). J Am Board Fam Med 2020;33(5):728-35.
s70. Shimada SL, Zocchi MS, Hogan TP, et al. Impact of Patient-Clinical Team Secure Messaging on Communication Patterns and Patient Experience: Randomized Encouragement Design Trial. J Med Internet Res 2020;22(11):e22307.
s71. Szilagyi PG, Albertin C, Casillas A, et al. Effect of Patient Portal Reminders Sent by a Health Care System on Influenza Vaccination Rates: A Randomized Clinical Trial. JAMA Intern Med 2020;180(7):962-70.
s72. Wijesundara JG, Ito Fukunaga M, Ogarek J, et al. Electronic Health Record Portal Messages and Interactive Voice Response Calls to Improve Rates of Early Season Influenza Vaccination: Randomized Controlled Trial. J Med Internet Res 2020;22(9):e16373.
s73. Willis TA, Collinson M, Glidewell L, et al. An adaptable implementation package targeting evidence-based indicators in primary care: A pragmatic cluster-randomised evaluation. PLoS Med 2020;17(2):e1003045.
s74. Dahne J, Player M, Carpenter MJ, et al. Evaluation of a Proactive Smoking Cessation Electronic Visit to Extend the Reach of Evidence-Based Cessation Treatment via Primary Care. Telemed J E Health 2021;27(3):347-54.
s75. Dzidowska M, Lee KSK, Conigrave JH, et al. Support for Aboriginal health services in reducing harms from alcohol: 2-year service provision outcomes in a cluster randomized trial. Addiction 2021;117(3):796-803.
s76. Gengiah S, Barker PM, Yende-Zuma N, et al. A cluster-randomized controlled trial to improve the quality of integrated HIV-tuberculosis services in primary healthcareclinics in South Africa. J Int AIDS Soc 2021;24(9):e25803.
s77. Hirsch-Moverman Y, Howard AA, Mantell JE, et al. Improving child tuberculosis contact identification and screening in Lesotho: Results from a mixed-methods cluster-randomized implementation science study. PLoS One 2021;16(5):e0248516.
s78. Howie AH, Klar N, Nash DM, et al. Printed educational materials directed at Ontario family physicians do not improve adherence to guideline recommendations for diabetes management: a pragmatic, factorial, cluster randomized controlled trial [ISRCTN72772651]. BMC Fam Pract 2021;22(1):243.
s79. Schneider JA, Young L, Ramachandran A, et al. A Pragmatic Randomized Controlled Trial to Increase PrEP Uptake for HIV Prevention: 55-Week Results From PrEPChicago. J Acquir Immune Defic Syndr 2021;86(1):31-37.
s80. Webster R, Usherwood T, Joshi R, et al. An electronic decision support-based complex intervention to improve management of cardiovascular risk in primary health care: a cluster randomised trial (INTEGRATE). Med J Aust 2021;214(9):420-27.

Additional file 5. Summary of single implementation strategies used in included trials

Single Implementation strategy Total = 24	N (%)	
**Reminders**	16	(66.7%)
**Education (including meetings, materials, trainings, workshops, educational outreach, etc.)**	5	(20.8%)
**CDSS**	3	(12.5%)

Additional file 6. Reported rationale themes with full examples

Rationale of using RHD in implementation trials

- **Improving quality of care**
  - "Collection of routine data could allow care quality to be monitored, and improved, by identifying patients who would benefit from a specific evidence-based treatment, fostering access to and monitoring retention in that treatment." [s22]
  - "The use of electronic health records (EHRs) offers potential for improving the quality of care for obese children and for accelerating the adoption of research evidence regarding childhood obesity screening and management. It also holds promise for establishing treatment benchmarks and for supporting patients and their clinical teams in care improvement" [s30]
  - "EHRs have the potential to increase adherence to guidelines, and it could be used to remind clinicians to document smoking status and deliver brief advice, prompt clinicians to prescribe cessation medications, and facilitate referrals to counseling." [s17]
  - "An EHR-based tool is available that can facilitate meaningful use documentation" [s43]
  - "The modern electronic health record (EHR) has previously been used to increase the likelihood of CHB screening via message prompts to providers 24 hours prior to appointments involving at-risk patients." [s48]
  - "Incorporating alerts into electronic medical records (EMRs) has proved effective for improving the practice of different preventive care" [s35]
- **Confirming findings**
  - "We evaluated the reliability of the trial results against routinely collected data from the health check management information system." [s39]
  - "The administrative database analysis offers partial validation for the assessment of adherence to medication." [s61]
- **Reducing research burden**
  - "It minimised the burden of research for practices and encouraged recruitment and it reduced the cost compared to trial-specific data collection." [s37]
  - "We were able to embed the intervention within the workflow of a primary care clinic, which resulted in high rates of participation by providers and a protocol that did not increase provider burden. " [s68]
  - "Embedding multi-arm, pragmatic trials of interventions aiming at implementing improved care or outcomes within routine operations of health systems can help to reduce research waste in implementation science by producing generalisable scientific results, and findings that directly improve patient care" [s61]
  - "Our trial was a cost-effective approach to reach a population of over 100 000 at-risk patients, costing less than £1 per patient." [s37]
- **Increasing efficiency**
  - "The conduct of trials using electronic health records as a means for increasing the speed and efficiency with which trials can be completed is a topic of growing importance." [s39]
  - "Registries can track influenza immunisation rates in recalled children and can increase the efficiency of reminder/recall by restricting subsequent mailings only to those patients who are not immunised after an initial recall letter." [s7]
  - "To maximise efficiency, the approach involved automated enrolment from an existing provincial registry, centralised delivery of the intervention, and use of routinely collected administrative data. " [s61]
- **Assessing outcomes**
  - Providing objective and reliable measures
    - "Outcomes were measured using routinely collected electronic health information, avoiding any distortions to usual care that might arise from data collection from physicians or patients." [s78]
    - "There was no reliable and practical biochemical method of verifying that an infant is breastfed, and strong concerns were voiced that seeking direct proof of breastfeeding (eg, through observation of a feed) would have a negative effect on the relationship between clinicians and women. We therefore used the method by which infant feeding status is recorded for the purposes of routine data collection in the UK's National Health Service. [54]
  - Ascertaining outcomes at lower cost
    - "An emerging trial methodology utilises routinely collected electronic health record sources to ascertain outcome data, at far lower cost than traditional methods of data collection." [s37]
  - Possessing improved accuracy
    - "These data provided objective measures that are less prone to inaccuracy than self reported healthcare data, which have consistently overestimate compliance with recommendations for preventive screening procedures." [s3]
    - "This claims-based measure has been shown to have improved accuracy and have higher correlation with biological markers of adherence than pill counts. It also reduces recall bias that may occur with self-reported adherence measures." [s32]
- **Identifying study samples**
  - "Registries pool data from multiple sources and can be used to target children most at risk and most likely to benefit from outreach." [s2]
  - "Billing data proved to be a powerful and efficient tool for identifying children who need influenza immunisation." [s7]
  - "Data contained in an electronic health record (EHR) can be used to identify candidates for risk-reducing interventions." [s26]
  - "For vaccination outreach, data recorded in the electronic health record through routine care delivery can inform real-time identification of unvaccinated populations." [s72]
  - "Data available in the EHR can be utilised to identify the appropriate target population for a smoking cessation intervention." [s74]
- **Assessing representativeness**
  - "We compared routinely collected child data on study participants and programme participants not taking part in the study to assess representativeness of the study sample." [s53]
  - "Use of EHR allowed for studying the natural process of care, studying a large number of patients, and having a population-based sample of patients and providers, rather than limiting our sample to those willing to be recruited into a study." [s31]
  - "CHSP data enabled us to assess maternal age and deprivation at population level." [s16]

Additional file 7. Reported facilitators and barriers with full examples

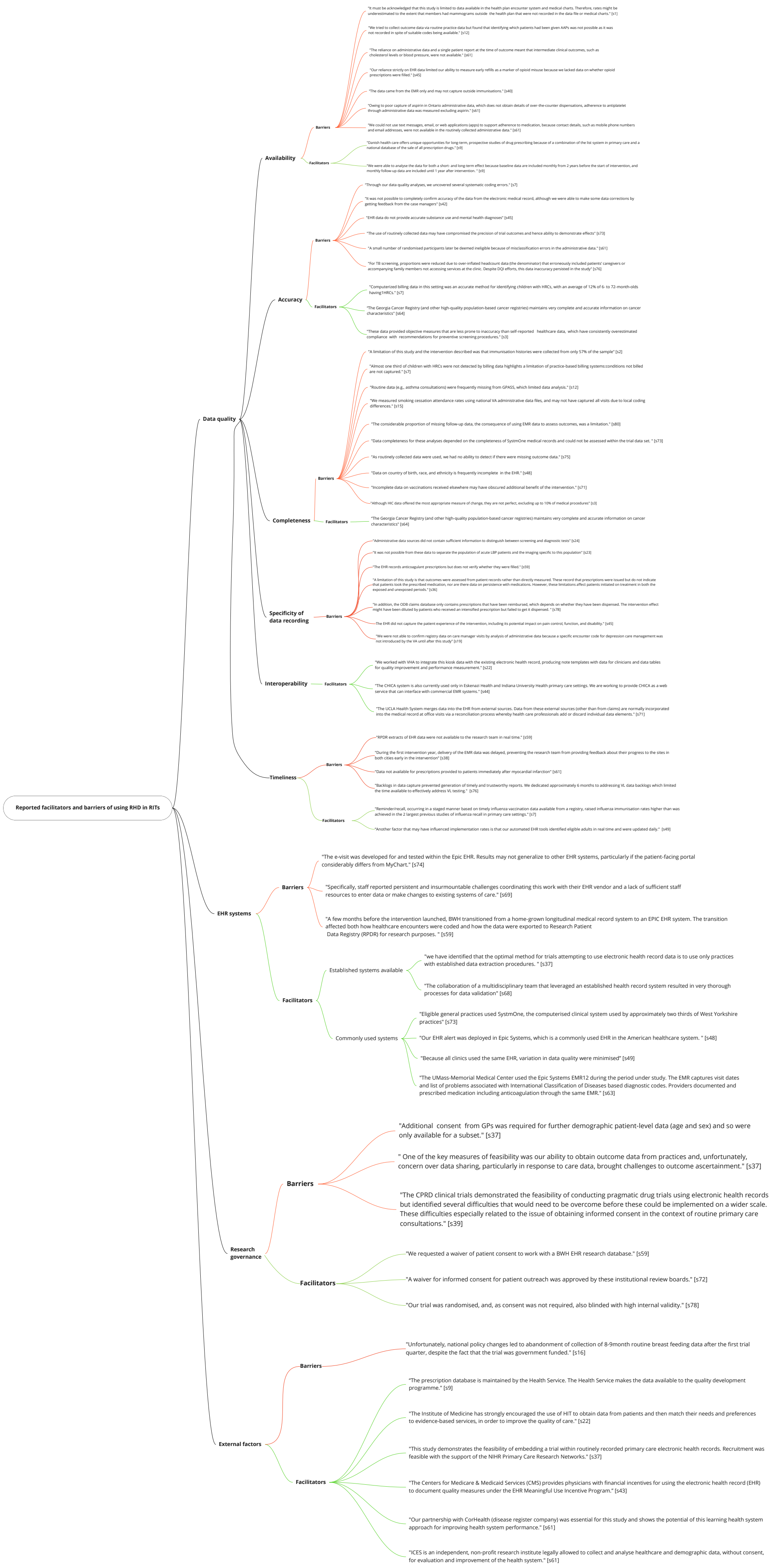

Supplement: Supplementary file 1 — Additional file 1: Additional file 1. PRISMA 2020 Checklist. Additional file 2. Search Strategy. Additional file 3. Description of implementation outcomes according to Proctor et al., 2010. Additional file 4. References for included trials. Additional file 5. Summary of single implementation strategies used in included trials. Additional file 6. Reported rationale themes with full examples. Additional file 7. Reported facilitators and barriers with full examples. [file 13012_2023_1300_MOESM1_ESM.pdf]
